# Drug-Induced Conformational Population Shifts in Topoisomerase-DNA Ternary Complexes

**DOI:** 10.3390/molecules19067415

**Published:** 2014-06-05

**Authors:** Nan-Lan Huang, Jung-Hsin Lin

**Affiliations:** 1 Division of Mechanics, Research Center for Applied Sciences, Academia Sinica, 128 Academia Rd., Sec. 2, Nankang, Taipei 115, Taiwan; E-Mail: nanlan@gate.sinica.edu.tw; 2 Institute of Biomedical Sciences, Academia Sinica, 128 Academia Rd., Sec. 2, Nankang, Taipei 115, Taiwan; 3 School of Pharmacy, National Taiwan University, 1 Jen-Ai Rd., Sec. 2, Taipei 10051, Taiwan

**Keywords:** topoisomerases II, molecular docking, molecular dynamics simulations, computer-aided drug design

## Abstract

Type II topoisomerases (TOP2) are enzymes that resolve the topological problems during DNA replication and transcription by transiently cleaving both strands and forming a cleavage complex with the DNA. Several prominent anti-cancer agents inhibit TOP2 by stabilizing the cleavage complex and engendering permanent DNA breakage. To discriminate drug binding modes in TOP2-α and TOP2-β, we applied our newly developed scoring function, dubbed AutoDock4^RAP^, to evaluate the binding modes of VP-16, *m*-AMSA, and mitoxantrone to the cleavage complexes. Docking reproduced crystallographic binding mode of VP-16 in a ternary complex of TOP2-β with root-mean-square deviation of 0.65 Å. Molecular dynamics simulation of the complex confirmed the crystallographic binding mode of VP-16 and the conformation of the residue R503. Drug-related conformational changes in R503 have been observed in ternary complexes with *m*-AMSA and mitoxantrone. However, the R503 rotamers in these two simulations deviate from their crystallographic conformations, indicating a relaxation dynamics from the conformations determined with the drug replacement procedure. The binding mode of VP-16 in the cleavage complex of TOP2-α was determined by the conjoint use of docking and molecular dynamics simulations, which fell within a similar binding pocket of TOP2-β cleavage complex. Our findings may facilitate more efficient design efforts targeting TOP2-α specific drugs.

## 1. Introduction

Topoisomerase is a prominent family of enzymes that manipulate DNA topology to release the supercoiling force introduced after DNA replication or transcription [1]. Eukaryotic type II topoisomerases (Top2) are multimeric enzymes that engender double-strand breaks of DNA. These enzymes resolve topological problems by transiently cleaving both strands of DNA to form a “cleavage complex”, through which another DNA segment can be passed over [2,3,4,5,6]. Human type IIA topoisomerase (TOP2) is well established as a target of many anticancer drugs [7]. TOP2-targeting dugs comprise natural and synthetic compounds of various chemical scaffolds. Several prominent TOP2-targeting drugs are used in the treatment of leukemia or solid tumors, including VP-16 (etoposide) of the podophyllotoxin group, *m*-AMSA (amascarine) of the acridines, and mitoxantrone of the anthraquinones.

VP-16 and other currently used TOP2-targeting anticancer drugs are suggested to be the “poisons” of both TOP2-α and TOP2-β [8,9,10], which increase the formation of covalent cleavage complex and hamper re-ligation of the cleaved DNA. TOP2-α poisoning was recognized to account primarily for the anticipated “therapeutic” cytotoxicity on cancer cells [11], while TOP2-β poisoning was proposed to be involved in the undesirable, drug-induced carcinogenesis [11] and cardiotoxocity [12]. The growing evidence in such distinct consequences could inspire drug developers to take TOP2-β as an “antitarget” [13] in designing new TOP2-targeting agents. Several crystal structures of the TOP2-DNA complex have been determined [14,15,16]. The growing knowledge on these structures can be promising in the design of new compounds that specifically target the cleavage complex of TOP2-α. Ma *et al.* conducted docking and DFT calculations on drug binding [17] using a structure of the ATPase domain [18] but not the DNA-cleaving domain of TOP2. Up to date there are no reported molecular simulations on the TOP2-DNA complexes.

In a previous study, we have implemented a variant of AutoDock4 scoring function, dubbed AutoDock4^RAP^ [19], using a well-established charge model for ligands, the Austin-model 1-bond charge correction (AM1-BCC) method [20,21]. AutoDock4 [22] has been extensively adopted in virtual screening of drug candidates and for prediction of ligand binding poses in protein pockets, and the AM1-BCC charge model has been used widely in molecular dynamics simulations with the AMBER force field [23,24]. Recently, we also have validated AutoDock4^RAP^ on evaluating the binding affinities of glycans to lectins [25]. The use of this robust scoring function may facilitate virtual screening on compounds with more diverse chemical scaffolds.

In the current study, we employed AutoDock4^RAP^ to evaluate the binding affinities of VP-16, *m*-AMSA, and mitoxantrone to the TOP2-DNA complexes. In the following context, the term “cleavage complex” will be used specifically to describe the enzyme-DNA complex, and the cleavage complex bound with small molecule drugs will be referred to as a “ternary complex”.

## 2. Results and Discussion

To evaluate the free energies of binding, we adopted three distinct approaches. The first one was to directly “rescore” the original binding mode in the crystallographic coordinates. In the second scheme, we allowed the ligand to move only in a restricted space and “refine” the ligand to a potential position with locally lowest binding free energy near to the crystallographic binding site. The third approach was to carry out a comprehensive search, rendering the ligand to have translational and torsional alterations, thereby “docking” the ligand to a larger space in the cleavage complex.

### 2.1. Molecular Docking Reproduced the Binding Mode of VP-16 in the Cocrystalized Cleavage Complex of TOP2-β

**Figure 1:**
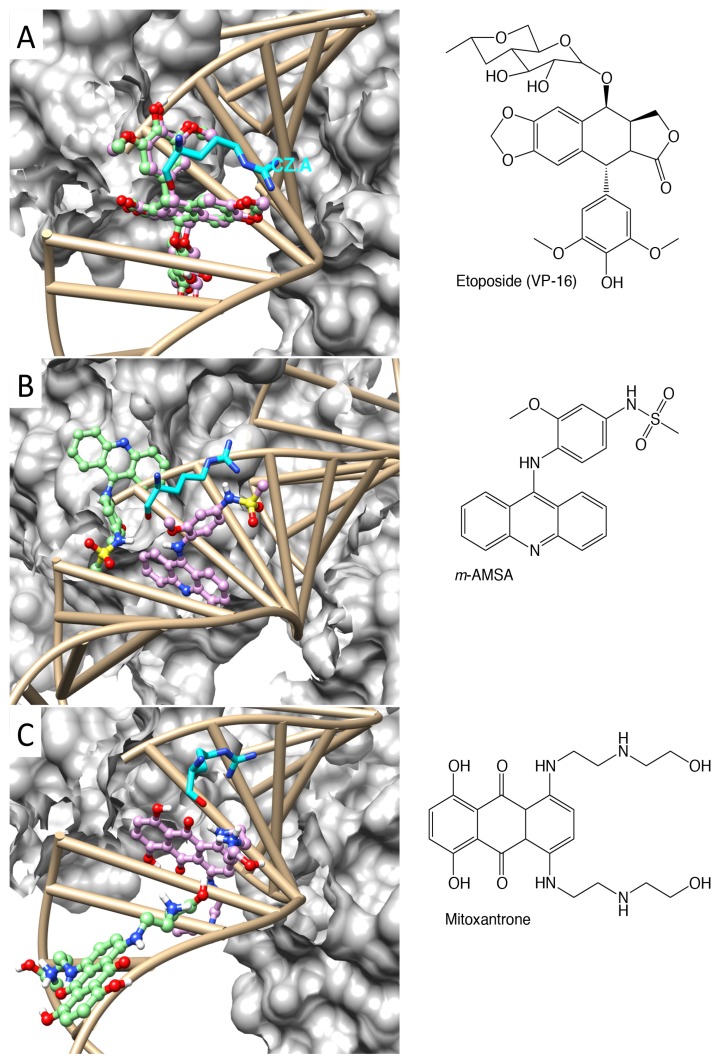
Crystallographic binding modes (pink) and docking poses (green) of VP-16 (**A**); *m*-AMSA (**B**); and mitoxantrone (**C**) in the cleavage complex of TOP2-β. The R503 in proximity to the ligands (cyan), the double-stranded DNA and the surface of one monomer of the dimerized proteins are shown.

A crystal structure of human TOP2-β in complex with double-stranded DNA and VP-16 was determined [14] with resolution of 2.16 Å (Figure S1, bottom). The two molecules of VP-16 were stabilized in the cleavage sites on both strands of DNA. In comparison with the quaternary conformation of a drug-free cleavage complex, insertion of VP-16 appeared to induce separation of the disjointed DNA ends [14,15,16]. Because it is the first ternary complex crystalized after the drug was stabilized in the cleavage complex, we started with this structure for molecular docking with the use of AutoDock4^RAP^. The grid box was centered on the geometric center of the DNA, with the grid spacing of 0.375 Å in each dimension and 100 × 100 × 100 grid points for each grid map, to enclose the two nicks on the cleaved DNA. Although the Mg^2+^ and the crystal water molecules were not taken into consideration in the molecular docking, the comprehensive search with the use of AutoDock4^RAP^ reproduced the crystallographic binding mode of VP-16 in the cleavage complex, with a root-mean-square deviation of 0.65 Å (Figure 1 and Table 1).

The crystal structures of human TOP2-β cleavage complexes bound with *m*-AMSA and mitoxantrone were characterized with a drug replacement procedure [16], with resolution of 2.70 and 2.55 Å, respectively. Similar to VP-16, the insertion of *m*-AMSA and mitoxantrone seemed to pull the cleaved DNA ends apart. However, when we applied the same approach to test our scoring function on these structures with a comprehensive search, the ligand molecules could barely be docked to their original geometric position in the crystals. The binding free energies of *m*-AMSA estimated with the “rescore” and the “refined” docking approaches were −2.68 and −3.17 kcal/mol, respectively. The binding free energies of mitoxantrone estimated with the “rescore” and the “refined” docking approaches were +1.26 and −1.20 kcal/mol, respectively. Given such weak affinities, these crystallographic binding modes, if once sampled in the docking process, may not be retained in the final list of free energy ranking after the comprehensive search.

The discrepancy in molecular docking of these structures may result from the sensitivity of the scoring function to subtle variations in the conformations of nucleic acid and amino acid residues induced by binding of different ligands. One such example can be demonstrated via fitting the electron density map (EDM) to the atomic model [26], which shows alternate conformations of R503 in the ternary complex of VP-16 (Figure S2). The alternate conformation of R503 used in molecular docking was the left one in Figure S2, which displayed a more intact “coating” in the EDM. In contrast, there are no defined alternate conformations of R503 in crystal structures of the complexes bound with *m*-AMSA and mitoxantrone. In the ternary complex bound with VP-16, R503 was indicated to have interaction with the polycyclic core and the aromatic E ring of the drug [14]. On the other hand, drug replacement resulted in marked variation of the R503 rotamer, as observed in the ternary complexes with *m*-AMSA and mitoxantrone [16] (Figure 1). Conformational change in the R503 rotamer was proposed to contribute to the transition in deoxyribose puckering and further to the drug-induced interference in DNA re-ligation [16]. To account for the potential induced-fit effects or population shifts of ligand binding on conformations of the enzyme and the DNA, we exploited molecular dynamics simulations on these ternary complexes of TOP2-β.

**Table 1:** Estimated binding free energies of VP-16, *m*-AMSA and mitoxantrone in the cleavage complex of TOP2-β.

Ligand	VP-16	*m*-AMSA	Mitoxantrone
Rescore (kcal/mol)	−14.3	−2.68	+1.26
Refined ∆G (kcal/mol)	−14.82	−3.17	−1.20
refined RMSD (Å)	0.62	0.64	1.07
Docking ∆G (kcal/mol)	−14.79	−7.53	−5.59
docking RMSD (Å)	0.65	8.01	13.50
Most probable ∆G (kcal/mol)	−12.51	−11.03	−10.72
estimated *K_i_* (nM)	0.68	8.21	13.98

### 2.2. Molecular Dynamics Simulations Revealed Conformational Changes of the Cleavage Complexes in Response to Ligand Binding

**Figure 2:**
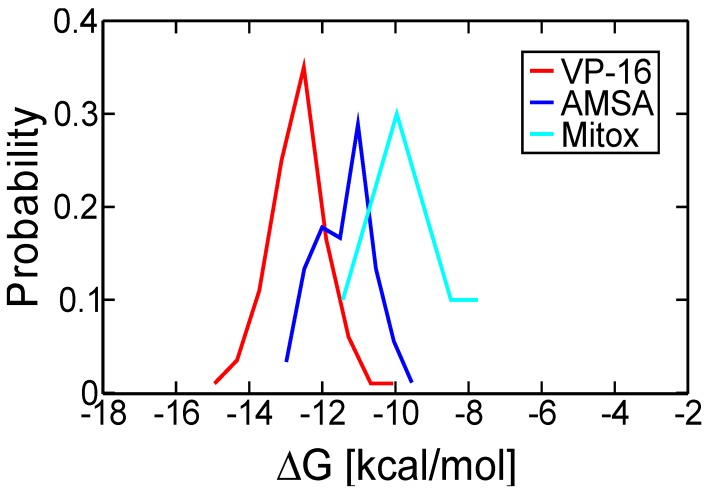
Free energy spectra of VP-16, *m*-AMSA and mitoxantrone for the TOP2-β cleavage complex.

We performed molecular dynamics (MD) simulations for 10 ns on the TOP2-β ternary complexes of *m*-AMSA and mitoxantrone. The Mg^2+^ ions and oxygen atoms of water molecules determined in the crystal structures were preserved in the simulations. Using the trajectories of the last 9-ns simulations, we obtained a conformation ensemble of each system and used the snapshots sampled every 100 ps to re-estimate the binding free energies, ∆G, using AutoDock4^RAP^. The estimated ∆G values of each system were analyzed to yield the probability density function of the binding free energies of the system, and the probability distribution was histogrammed against the ∆G values of the corresponding drug molecule, giving rise to the free energy spectrum [27] of the drug for the TOP2-β cleavage complex (Figure 2).

From the free energy spectrum of a ligand, the ∆G value with the highest probability was considered the most probable energy state as the drug molecule was bound in the nick of DNA in the cleavage complex (Table 1). The most probable binding free energies of *m*-AMSA and mitoxantrone from MD simulations are much more negative than the predicted binding free energies that rank top in the molecular docking with the use of crystal structures (Table 1). In addition, if we go leftward from the peak values along the free energy spectra of *m*-AMSA and mitoxantrone, the ending points signalize that we could also sample the conformations that occurs at times during the simulations, in which the drugs have even higher binding affinities to the cleavage complexes.

As we retrieved the dihedrals of the side chain of R503 from the MD trajectories, we made another intriguing finding. R503 is suggested to play an important role in stabilizing VP-16 in the cleavage complex [14,16]. We measured the four dihedrals of its side chain in the crystal structure (Table S1) and in the MD snapshots (Figure S3). In the ternary complex with VP-16, these dihedrals of R503 in the MD snapshots are similar to that in the crystal, implying a “stabilized” configuration of R503 rotamer in the presence of VP-16.

When the cleavage complex is bound with *m*-AMSA, the χ_1_ of R503 stay mainly around −70 degree, and the χ_2_ stay mainly around 180 degree (Figures S3A,B), similar to the complex bound with VP-16. Although the χ_3_ and χ_4_ in the ternary complex of *m*-AMSA have peak values different from those in the complex of VP-16, the χ_1_–χ_3_ configurations of the ternary complex of *m*-AMSA during MD simulations are in agreement with its crystallographic configuration, indicating a different conformation of R503 rotamer favorable for binding of *m*-AMSA. The deviation in χ_4_ of crystal structures and MD snapshots might echo the suboptimal binding affinity of *m*-AMSA, as revealed in the free energy spectra (Figure 2).

Furthermore, the cleavage complex bound with mitoxantrone had a disparate crystallographic conformation of the R503 rotamer. We also observed the preferred χ_1_ turned to −170 degree, with a minor peak value around −100 degree (Figure S3C), from the MD snapshots. The χ_2_ and χ_3_ both had multiple peak values, and the χ_4_ appeared to disperse without a well-defined peak value. Such variable distribution of these dihedrals could reflect the incongruity of mitoxantrone and the gate-keeping side chain of R503, probably leading to a lower binding affinity of mitoxantrone with the TOP2-β cleavage complex, which is in accord with the results seen in the free energy spectra (Figure 2).

**Figure 3:**
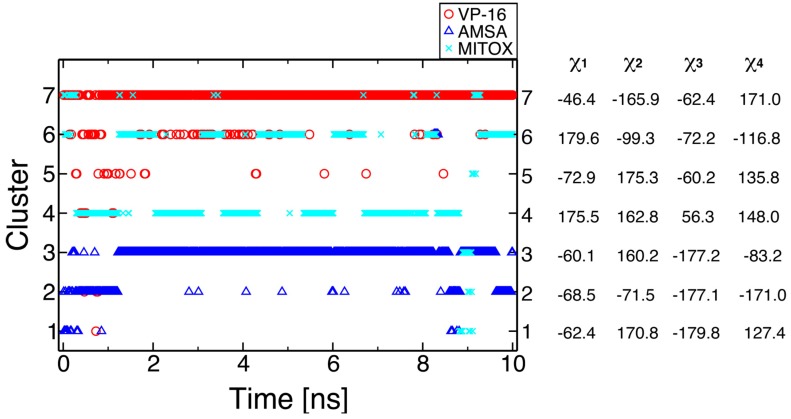
Clustering of the R503 rotamers in TOP2-β cleavage complexes bound with VP-16 (red), *m*-AMSA (blue) and mitoxantrone (cyan). Clusters 1 and 5 correspond to the crystal conformations of *m*AMSA-bound and mitoxantrone-bound complex, respectively. Cluster 7 corresponds to the crystal alternate conformation of VP16-bound complex used in the simulation; Cluster 2 corresponds to the other alternate conformation. The dihedrals χ_1_–χ_4_ of the cluster centroids are also shown.

To gain a panoramic view of the dynamic behavior of R503 rotamer, we conducted cluster analysis based on root-mean-square deviations of these dihedrals. The ternary complex with VP-16 adopts a dominating configuration of R503 rotamer, Cluster 7, which is also the configuration corresponding to the crystallographic conformation (Figure 3). R503 of the complex with *m*-AMSA tends to be stabilized in a configuration, Cluster 3, which is different from its crystallographic conformation, Cluster 1, suggesting an induced-fit effect upon drug binding. The complex with mitoxantrone, however, displays a bimodal distribution of the R503 rotamer (Figure 3, cyan symbols). This is consistent with the distribution of its four dihedrals (Figure S3C) and reiterates the unsettled R503 rotamer in the presence of mitoxantrone. A remarkable finding is that although we only took one alternate crystal conformation in VP16-bound complex as the starting point of simulation, a configuration corresponding to the other alternate conformation, Cluster 2 (Figure 3, red symbols), could be sampled in the simulations (RMSD of cluster centroid to the unused conformation: 0.481 Å; RMSD to the used conformation: 0.889 Å). In summary, MD simulations revealed the population shifts in the conformations of TOP2-DNA complexes upon drug binding, as reflected in the free energy spectra and R503 rotamer configurations.

### 2.3. Discriminating the High-Affinity Binding Mode of VP-16 in the Cleavage Complex of TOP2-α Using Free Energy Spectra

Ever since the discovery that the anticipated cytotoxicity of VP-16 is primarily TOP2-α-dependent while the unwanted drug-related carcinogenesis is attributed to its TOP2-β poisoning activity [11], the importance of developing TOP2-α-specific anticancer drugs has drawn increasing attention. However, up to date there is no reported crystal structure with small molecules bound in the DNA-cleaving complex of human TOP2-α. A crystal structure of human TOP2-α in complex with double-stranded DNA was determined [15] with resolution of 2.90 Å. We first used the biological assembly of this structure, which comprises the dimerized cleavage core of the enzyme and the double-stranded DNA with a nick on each strand, in molecular docking, with VP-16 as the ligand. In TOP2-β cleavage complexes stabilized by the three drugs, insertion of the planar, polycyclic moieties induced separation of the disjointed DNA ends, and the protein monomers were observed to slide, or swing, apart from each other [14,15,16] (Figure S1). In contrast to the drug–introduced amendment to the quaternary conformation of TOP2-β ternary complex, the crystalized enzyme-DNA complex of TOP2-α seemed to mimic an earlier step during the cleaving process, where the phosphodiester bonds between the nucleotides were just broken without apparent changes in the atomic coordinates of the nucleotides (Figure S1). There is no sufficient space in the nicks to accommodate the ligands. In spite of this observation, we selected the best three predicted binding modes of VP-16 according to the free energy ranking, and we conducted MD simulations on these ternary complex systems of TOP2-α. Based on the observation that, for the ternary complex of TOP2-β and mitoxantrone, the free energy spectrum constructed using snapshots of 3-ns simulations revealed a pattern comparable to that using snapshots of 10-ns simulations (not shown), we conducted the MD simulations on each TOP2-α system for 3 ns and used the snapshots sampled every 10 ps from the last ns for the re-estimation of binding free energies.

**Figure 4:**
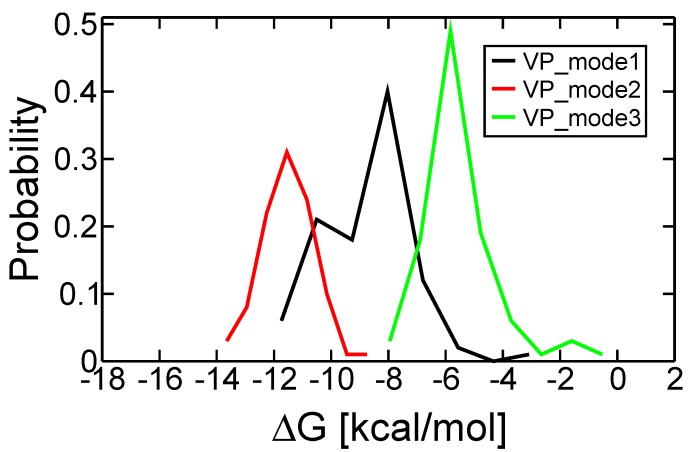
Free energy spectra of the best three binding modes of VP-16 in the TOP2-α cleavage complex.

**Figure 5:**
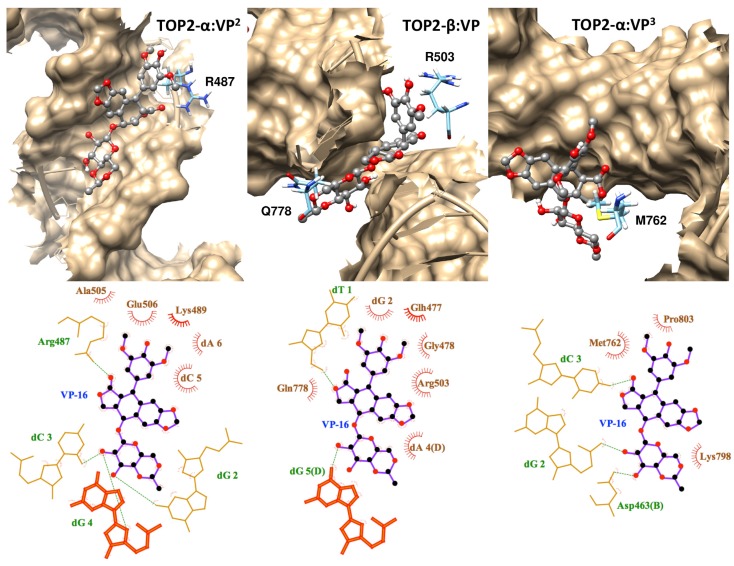
Interaction diagrams of VP-16 with the cleavage complexes of TOP2-α and TOP2-β (**middle**). Both the high-affinity (**left**; VP^2^) and the low-affinity (**right**; VP^3^) binding modes of VP-16 in the ternary complex of TOP2-α are shown. Upper panel, molecular graphics with labels on the R487/R503 and M672/Q778 of the enzymes and molecular surface of the cleaved DNA. Lower panel, the 2-dimensional maps are plotted by fitting the orientation of the ligand.

The docking free energies predicted by AutoDock4^RAP^ were comparable among the selected binding modes of VP-16 (Table 2). To our surprise, the free energy spectra constructed using the MD snapshots are distinct from one another (Figure 4 and Table 2). We selected the system with the lowest peak value of free energy spectrum, mode 2, and used its representative binding pose, one MD snapshot with such a ∆G value, to analyze the interactions between VP-16 and the cleavage complex using LigPlot+ [28]. We also conducted MD simulations on the ternary complex of VP-16 and TOP2-β and used its representative binding mode in the analyses (Figure 2). In the TOP2-α ternary complex, a nitrogen atom in the side chain of R487, the counterpart residue of R503 in TOP2-β, is at a distance and orientation that could favor hydrogen bonding with the carbonyl oxygen on the tetracyclic aglycone core of VP-16 (Figure 5). Although VP-16 was not stabilized in the DNA cleavage site of the TOP2-α complex, the interaction diagrams of the two systems reveal similar patterns involving both amino acid and nucleic acid residues surrounding the cleavage site of DNA.

**Table 2:** Estimated binding free energies of VP-16 in the cleavage complex of TOP2-α.

Initial Binding Mode	1	2 ^a^	3
Docking ∆G (kcal/mol)	−10.43	−10.01	−9.58
Most probable ∆G (kcal/mol)	−8.07	−11.54	−5.83
Estimated K_i_	1.21 µM	3.48 nM	52.88 µM

^a^ The most probable energy state of mode 2 was used as the representative binding mode.

A mismatch in the diagrams is Q778 of TOP2-β, which locates in the opposite side of the DNA. In this case we could not rule out the possibility that the mismatch result from the geometric hindrance of the DNA since the ligand was positioned to the viewing face in the cleavage complex of TOP2-α (Figure 5). Nevertheless, based on previous structural alignments, M762 of TOP2-α was proposed to be the corresponding residue for Q778 of TOP2-β [14,15]. The existence of these unconserved counterpart residues could exemplify the potential target residues for designing new drugs that specifically bind to the cleavage complex of TOP2-α. The potential contribution of M762 in ligand binding to the TOP2-α cleavage complex may be investigated by using MD simulations with enhanced sampling techniques.

A plausible way to tailor such agents could be grounded in discriminating a set of amino acid residues different from the residues expected to have interactions with the drugs currently in use, hopefully including M762 of TOP2-α. Interestingly, in mode 3, another binding mode provided by molecular docking, the VP-16 molecule was predicted to have van der Waals interaction with M762 of TOP2-α (Figure 5). The interaction diagram of this low-affinity binding mode of VP-16 could facilitate recognition of such a disparate group of residues for designing novel TOP2-α-targeting agents. New structures of TOP2-α ternary complex [29], in which the ligands are stabilized in the DNA cleavage sites, will also provide valuable information that can be exploited in conjunction with MD simulations to address the issue.

## 3. Experimental

### 3.1. Protein Models of TOP2-α and TOP2-β

The crystallographic coordinates of human TOP2-β ternary complexes (PDB code: 3QX3, 4G0U, 4G0V) were used as template structures. Each ternary complex of TOP2-β comprises the dimerized cleavage core (S445-D1201) of the enzyme, the double-stranded DNA with a nick on each strand, and two molecules of a drug (VP-16, *m*-AMSA or mitoxantrone). The coordinates of missing residues in the loops were assigned using MODELLER 9.11, with the nucleic acid and ligand residues copied as rigid bodies into the built model. Atomic coordinates of the newly built model were mapped to the crystallographic coordinates using structural alignment. Subsequently, only the mapped coordinates of amino acid residues were retained in conjunction with crystallographic coordinates of the DNA, the ligands, the Mg^2+^ ions and oxygen atoms of crystal water molecules, generating a model system of each complex for molecular dynamics (MD) simulations.

The biological assembly of human TOP2-α cleavage complex comprises the dimerized cleavage core (K431-L1193) of the enzyme and the double-stranded DNA with a nick on each strand (PDB code: 4FM9). Up to date there is no reported crystal structure with small molecules bound in the DNA-cleaving complex of human TOP2-α. To avoid adding excessive missing residues in the N-terminal and C-terminal loop regions, only 660 residues (N433-E1092) of the TOP2-α isozyme were used in model building. The model system was generated as described above, except that no ligands were used. Instead, molecular docking was used to provide initial atomic coordinates of VP-16 in the cleavage complex for MD simulations.

### 3.2. Molecular Docking with the Use of AutoDock4^RAP^

We applied AutoDock 4.0 [22] and our newly developed scoring function for protein-ligand interactions, dubbed AutoDock4^RAP^ [19], to assess the binding affinities of the drugs to the cleavage complex of TOP2-β, as well as to provide initial binding modes of VP-16 to the TOP2α-DNA complex for subsequent analyses. Protonation of the ligands were carried out using openbabel [30], and protonation of the protein-DNA complexes were conducted using PDB2PQR [31,32]. The atomic charges of ligands were calculated with the Austin-model 1-bond charge correction (AM1-BCC) methods [20,21], and the atomic charges of proteins were retrieved from the AMBER parm99SB force field parameters [33,34,35]. For each enzyme-DNA complex, the grid box was centered on the geometric center of the DNA, with the grid spacing of 0.375 Å in each dimension and 100 × 100 × 100 grid points for each grid map. The rapid docking served as the initial filtering approach to screen a myriad of binding poses to a limited set, and the best few predicted binding modes of each compound were further analyzed with molecular dynamics simulations.

### 3.3. Molecular Dynamics Simulations, Free Energy Spectra, and Analyses on R503 Rotamer

Molecular dynamics simulations were carried out using the PMEMD module of AMBER 12 package [36], with the use of the particle-mesh Ewald (PME) method for calculating the full electrostatic interactions of a periodic box in the macroscopic lattice of repeating images. Sampling of individual snapshots from MD trajectories, retrieval of dihedrals of R503 rotamer, and clustering were carried out using the cpptraj module [37,38]. The snapshots were subjected to re-estimation of the binding free energies with the use of AutoDock4^RAP^. Free energy spectra and probability distribution of dihedrals of R503 rotamer were constructed using in-house programs and scripts. Details of computation methods are inscribed in supplementary information.

### 3.4. Interaction Diagrams

The representative binding modes of the compounds to the enzyme-DNA complexes were subjected to analyses of ligand-protein interactions using LigPlot+ [28]. The Mg^2+^ ions and water molecules were not included in the analyses. Molecular graphics of the representative binding modes were generated using UCSF chimera [39], and protein residues that have interactions with the ligand were labeled in the graphics according to the results of LigPlot+.

## 4. Conclusions

Molecular dynamics simulation of TOP2-β in complex with VP-16 confirmed the X-ray crystallographic binding mode. In contrast, the conformations of R503 of TOP2-β in complex with m-AMSA and mitoxantrone deviates from their original crystallographic conformations, indicating a relaxation dynamics from the conformations determined with the soaking procedure for preparing the *holo* protein crystals. The binding mode of VP-16 in the cleavage complex of TOP2-α was determined by docking and molecular dynamics simulations, which fell within a similar binding pocket of TOP2-β cleavage complex. Our results may facilitate more efficient designing toward TOP2-α specific drugs.

## Supplementary Materials

Supplementary materials can be accessed at:http://www.mdpi.com/1420-3049/19/6/7415/s1.
