# Supplementary material for: Drug-Induced Conformational Population Shifts in Topoisomerase-DNA Ternary Complexes
Source: Molecules. 2014 Jun 5;19(6):7415–28. doi: 10.3390/molecules19067415 (PMC6272011; doi:10.3390/molecules19067415)
Supplement: Supplementary file 1 [file molecules-19-07415-s001.pdf]

Supplementary Materials

Figure S1. Cleavage complexes (**left**) and the cleaved DNA (**right**, enlarged) in the crystal structures of TOP2-α (PDB code: 4FM9) and TOP2-β (PDB code: 3QX3). The monomeric units of the dimerized enzyme were shown in distinct colors. The Mg^{2+}, oxygen atoms of water molecules and the two molecules of VP-16, bound in the TOP2-β cleavage complex, were also shown.

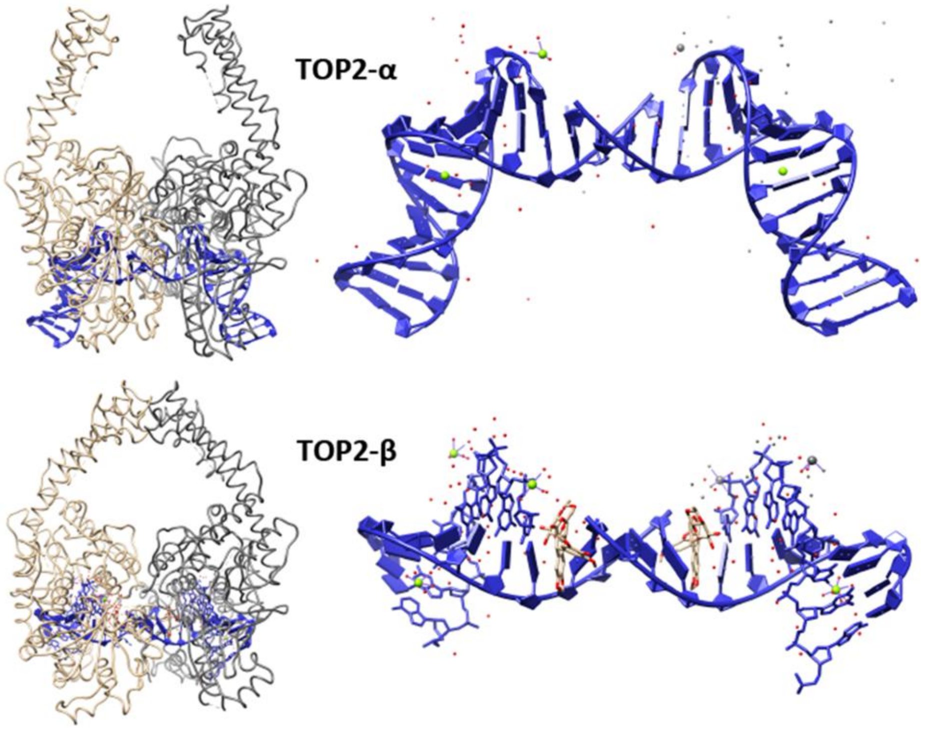

Figure S2. Electron density map (EDM) showing alternate crystallographic conformations of R503 in the ternary complex of VP-16. The EDM of side chain atoms of R503 is displayed at the contour level of 0.09 with the mesh scheme colored in cyan.

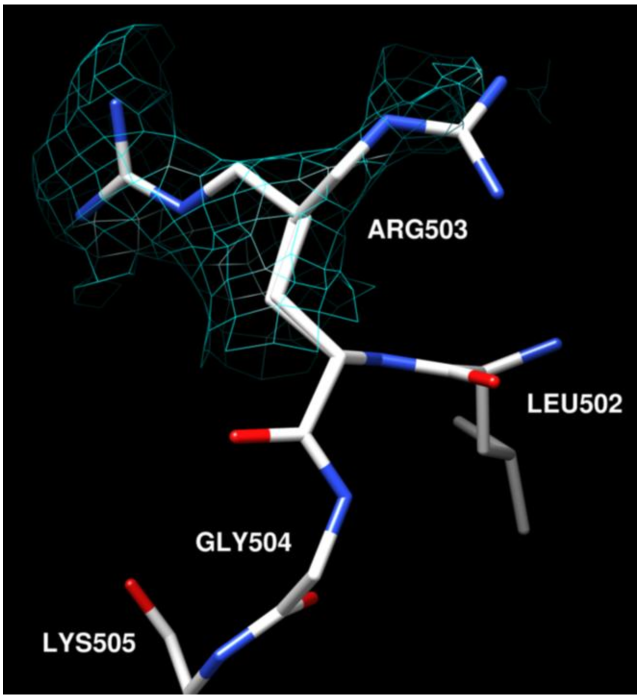

Figure S3. Probability distribution of the dihedrals, χ_1–χ_4, for the side chain of R503 in the TOP2-β cleavage complex bound with VP-16 (**A**); *m*-AMSA (**B**); and mitoxantrone (**C**).

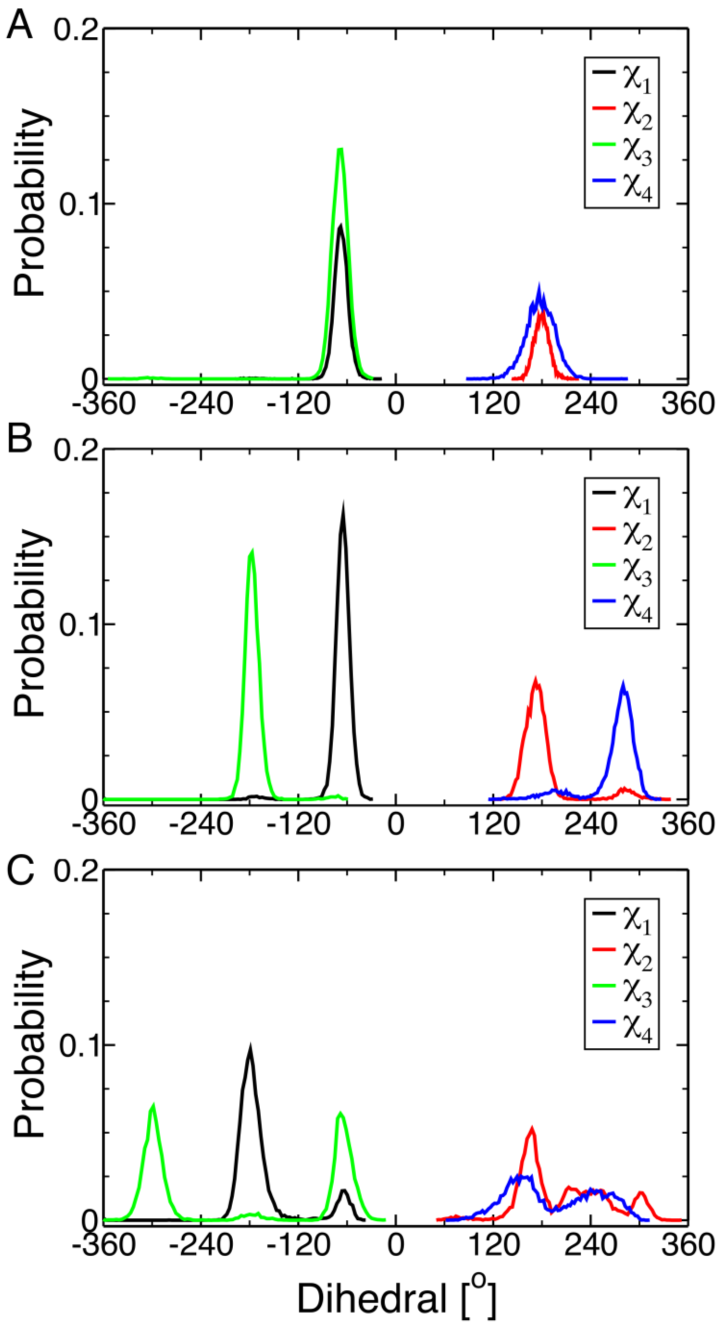

Table S1. Side chain dihedrals (°) of R503 in the crystal structures of TOP2-β cleavage complex bound with VP-16, *m*-AMSA and mitoxantrone. Dihedrals were measured using UCSF chimera, defined in the range of −180 to 180 degree.

Complex	χ_1	χ_2	χ_3	χ_4
**VP-16**	−55.9	168.8	−43.9	−171.6
***m*-AMSA**	−88.2	169.6	−166.4	177.4
**Mitoxantrone**	−91.3	−157.7	−84.7	149.5

Table S2. Side chain dihedrals (°) of R503 in the cluster centroids of TOP2-β cleavage complexes. Clustering and dihedral measurements were conducted using cpptraj program. Dihedrals are defined in the range of −180 to 180 degree.

Cluster Centroids	χ_1	χ_2	χ_3	χ_4
**1**	−62.4	170.8	−179.8	127.4
2	−68.5	−71.5	−177.1	−171.0
**3**	−60.1	160.2	−177.2	−83.2
**4**	175.5	162.8	56.3	148.0
**5**	−72.9	175.3	−60.2	135.8
**6**	179.6	−99.3	−72.2	−116.8
**7**	−46.4	−165.9	−62.4	171.0

1. Supplementary Computation Methods

1.1. Molecular Dynamics Simulations

Molecular dynamics simulations were carried out using the PMEMD module of AMBER 12 package [1], with the use of the particle-mesh Ewald (PME) method for calculating the full electrostatic interactions of a periodic box in the macroscopic lattice of repeating images. The topology files and parameter files were prepared using the LEaP module. Each system, namely a protein bound with a compound, was solvated with water molecules in a cubic box, with the buffer distance of 12.0 Å to the box boundary and the closeness parameter of 0.75 Å, using TIP3P explicit solvent model [2]. Ions were supplemented using the addIons command to simulate an environment of 150 mM sodium chloride aqueous solution. Energy minimizations were carried out with restraints on atoms of the proteins and the compounds, as well as the Mg^{2+} and the oxygen atoms of the crystal water molecules. The systems were heated to 300 K and maintained constant using the weak-coupling algorithm with the coupling time constant, τ_T, of 0.2 ps. System pressures were regulated using isotropic position scaling, and the Berendsen barostat was used with the relaxation time, τ_P, of 2 ps. NPT (isothermal-isobaric) simulations were conducted to equilibrate the system densities, without restraints on atoms, for 20 ps with the time step of 1 fs. Afterwards, production runs were carried out with the time step of 2 fs and with SHAKE constraints [3] on the bonds involving hydrogen. Individual snapshots were sampled from the trajectories of the production simulations using the cpptraj module [4]. These snapshots were subjected to re-estimation of the binding free energies with the use of AutoDock4^{RAP}.

1.2. Construction of the Binding Free Energy Spectra

The estimated binding free energies (ΔG) of the MD snapshots for each system were analyzed using an in-house program to yield the free energy spectra, which are the probability density functions of the binding free energies of the system. The number of bins, *k*, for each system in histogram analysis was determined by the following formula derived from Sturges' rule [5]:

$$k = \lceil 1 + \log_2 n \rceil \tag{1}$$

where $\lceil \ \rceil$ is the ceiling operator and *n* is the number of snapshots of that system. The free energy spectra [6] of the compounds for TOP2-α and TOP2-β were constructed, respectively. From the free

energy spectra of a compound, the ΔG value with the highest probability was considered the most probable energy state as the compound was bound in the binding pocket of the kinase, and one MD snapshot with such ΔG value was selected as the representative binding mode of the compound to the kinase. The most probable energy state of each compound-kinase system was translated into an estimated K_i value using the recalibrated Gibbs free energy formula [7]:

$$\Delta G = -RT \ln K_i \quad (2)$$

where *R* is the ideal gas constant in units of J/mol K, and *T* is the temperature in kelvin.

1.3. Probability Distributions of the Dihedrals of R503

The atoms used to define the dihedrals, χ_1–χ_4, for the side chain of Arg are as follows: χ_1, N-Cα-Cβ-Cγ; χ_2, Cα-Cβ-Cγ-Cδ; χ_3, Cβ-Cγ-Cδ-Nε; χ_4, Cγ-Cδ-Nε-Cζ. These dihedrals of R503 in the chain A of TOP2-β were retrieved from the last 9-ns MD trajectories using the cpptraj module, yielding the dihedral values of the 9000 snapshots. The probability distributions of the dihedrals were constructed as described in the previous section, with the number of bins, *k*, equal to 100.

1.4. Clustering of R503 Rotamer

Clustering of 3000 snapshots, which comprise 1000 snapshots sampled from the trajectory of each of the three TOP2-β ternary complexes, was carried out using cpptraj module [8]. The agglomerative average-linkage algorithm was applied, and the best-fit coordinate RMSDs of non-hydrogen atoms in the side chain of R503 between the snapshots were used in the distance matrix. The iterations stopped when the minimum distance between clusters is greater than 0.4 and gave rise to seven clusters. RMSD of these atoms in each cluster centroid to the four crystallographic R503 rotamer conformations, which include the conformations of the ternary complexes with *m*-AMSA and mitoxantrone, as well as 2 alternate conformations of the ternary complex with VP-16, were calculated. The cluster with the smallest RMSD of its centroid to a crystal conformation is considered the corresponding R503 rotamer configuration of that crystal conformation.

References

1. Case, D.A.; Babin, V.; Berryman, J.T.; Betz, R.M.; Cai, Q.; Cerutti, D.S.; Cheatham, T.E., III; Darden, T.A.; Duke, R.E.; Gohlke, H.; *et al. AMBER 12*; University of California: San Francisco, CA, USA, 2012.
2. Jorgensen, W.L.; Chandrasekhar, J.; Madura, J.D.; Impey, R.W.; Klein, M.L. Comparison of simple potential functions for simulating liquid water. *J. Chem. Phys.* **1983**, *79*, 926–935.
3. Ryckaert, J.-P.; Ciccotti, G.; Berendsen, H.J.C. Numerical integration of the cartesian equations of motion of a system with constraints: Molecular dynamics of n-alkanes. *J. Comput. Phys.* **1977**, *23*, 327–341.
4. Roe, D.R.; Cheatham, T.E. PTRAJ and Cpptraj: Software for processing and analysis of molecular dynamics trajectory data. *J. Chem. Theory Comput.* **2013**, *9*, 3084–3095.
5. Sturges, H.A. The choice of a class interval. *J. Am. Stat. Assoc.* **1926**, *21*, 65–66.

6. Lin, J.H.; Perryman, A.L.; Schames, J.R.; McCammon, J.A. The relaxed complex method: Accommodating receptor flexibility for drug design with an improved scoring scheme. *Biopolymers* **2003**, *68*, 47–62.
7. Gibbs, J.W. A method of geometrical representation of the thermodynamic properties of substances by means of surfaces. *Trans. Conn. Acad. Arts Sci.* **1873**, *2*, 382–404.
8. Shao, J.; Tanner, S.W.; Thompson, N.; Cheatham, T.E. Clustering molecular dynamics trajectories: 1. Characterizing the performance of different clustering algorithms. *J. Chem. Theory Comput.* **2007**, *3*, 2312–2334.

© 2014 by the authors; licensee MDPI, Basel, Switzerland. This article is an open access article distributed under the terms and conditions of the Creative Commons Attribution license (http://creativecommons.org/licenses/by/3.0/).
